# Dynamic QoS Prediction Algorithm Based on Kalman Filter Modification

**DOI:** 10.3390/s22155651

**Published:** 2022-07-28

**Authors:** Yunfei Yan, Peng Sun, Jieyong Zhang, Yutang Ma, Liang Zhao, Yueyi Qin

**Affiliations:** 1 Information and Navigation College, Air Force Engineering University, Xi’an 710077, China; yyf435382221@163.com (Y.Y.); dumu3110728@126.com (J.Z.); tuzhong0804@163.com (Y.M.); op33561165565@163.com (L.Z.); 2 School of Economics and Management, Chang’an University, Xi’an 710077, China; 13335377128@163.com

**Keywords:** service recommendation, Quality of Service, service computing, deep learning

## Abstract

With the widespread adoption of service-oriented architectures (SOA), services with the same functionality but the different Quality of Service (QoS) are proliferating, which is challenging the ability of users to build high-quality services. It is often costly for users to evaluate the QoS of all feasible services; therefore, it is necessary to investigate QoS prediction algorithms to help users find services that meet their needs. In this paper, we propose a QoS prediction algorithm called the MFDK model, which is able to fill in historical sparse QoS values by a non-negative matrix decomposition algorithm and predict future QoS values by a deep neural network. In addition, this model uses a Kalman filter algorithm to correct the model prediction values with real-time QoS observations to reduce its prediction error. Through extensive simulation experiments on the WS-DREAM dataset, we analytically validate that the MFDK model has better prediction accuracy compared to the baseline model, and it can maintain good prediction results under different tensor densities and observation densities. We further demonstrate the rationality of our proposed model and its prediction performance through model ablation experiments and parameter tuning experiments.

## 1. Introduction

### 1.1. Background and Motivation

With the widespread use of Service-Oriented Architecture (SOA) in software development efforts, the reusability and interoperability of services have been greatly enhanced while promoting continuous progress in research on service clustering and classification [1,2,3], service recommendation [4,5,6], and service combination [7,8,9]. With the popularity of SOA architectures, the number of available web services has grown exponentially, which has resulted in a large number of functionally identical or similar services in the network. Quality of Service (QoS) is a set of service non-functional evaluation metrics widely used nowadays, through which the merits of network services with the same functions can be easily measured. It has become an important challenge to construct high-quality services that meet the non-functional needs of users from a large number of services with the same function [10].

For example, when using a service or building a combination of services, users can usually select a large number of candidates whose functionality meets the requirements of the user, but it is difficult to visualize whether the QoS properties of these services meet the requirements, which contains two main aspects of the problem.

Objectively, services are usually deployed in cloud servers, and users invoke these services remotely through the network. Therefore, the QoS felt by the client will be affected by both the state of the service itself and the network environment, such as the service operation state, service load, network fluctuation situation, and network congestion, making the QoS a real-time changing value. This is also the reason why different users get different QoS experiences for the same service. Therefore, it is difficult for users to obtain a credible QoS directly.

Subjectively, it is a costly task for users to actively evaluate the QoS value of a service. On the one hand, service providers usually charge for executing service invocations, which can cause significant financial expenses; on the other hand, continuous observation of all compliant services for the purpose of QoS assessment consumes a lot of time and resources. Therefore, it is often difficult for users themselves to evaluate the service QoS by means of service invocations.

In summary, at a high cost, users can usually only invoke a limited number of services with sparse QoS in order to comprehensively evaluate the QoS properties of services and avoid consuming high costs. How to predict the missing QoS becomes the core problem of building high-quality web services today.

### 1.2. Related Works

Prediction problems are studies in which people speculate about the trends that will emerge in the future based on the development patterns of historical things. Research on prediction problems is developing rapidly in a wide range of fields such as epidemiology [11,12], network science [13,14], engineering management [15,16], and cloud computing [17,18]. In the field of service recommendation, current research on QoS prediction problems can be divided into two categories, static QoS prediction methods [19,20,21,22,23] and dynamic QoS prediction methods [24,25,26,27,28,29,30,31].

Static QoS prediction problems often perform QoS prediction under fixed time slices according to the contextual relationships between users and services such as location information and network information. Studies based on collaborative filtering (CF) methods usually fall into this category of problems. CF-based QoS prediction methods can be classified as neighborhood-based and model-based. The neighborhood-based CF algorithm assumes a stable similarity relationship between users or services. The similarity between users can be calculated by using historical QoS values as features and filling in the missing QoS values with historical information between similar users. Shao et al. [19] first used a collaborative filtering approach for QoS prediction. This study mainly constructs the QoS matrix of users and services, obtains a similarity relationship between users, and finally predicts the missing QoS of the target users. Zheng et al. [20], based on the previous work, proposed the method of integrating user similarity and service similarity for QoS prediction, which effectively improves the accuracy of QoS prediction.

Model-based CF algorithms acquire the implicit relationships between users and services by building specific models to predict the desired QoS values. Xia et al. [21] first extracted multi-source features through a combination of matrix decomposition and neural networks and improved the prediction capability of sparse QoS data by deep neural networks for feature learning. Zou et al. [22] proposed a domain-integrated deep matrix decomposition algorithm, which improves the ability to obtain implicit features of users and services through the fusion of deep neural networks and matrix decomposition. Nguyen et al. [23] proposed an attention probability matrix model, which learns latent features by introducing a neural attention network on the basis of probability matrix decomposition and proposes a neural network architecture to learn latent features of services.

However, in the real environment, different users will access the same service at different times. At the same time, the QoS value of the same service will change over time, which will make the QoS value of the service invoked by the user always in dynamic change. Having the ability to predict QoS changes for future time slices would better meet the demand of users for high-quality services.

A dynamic QoS prediction algorithm aims to predict the future QoS development pattern based on historical QoS data features, and this method is now becoming a new research hotspot. The main research directions of current dynamic QoS prediction algorithms can be divided into feature engineering-based methods and deep learning-based methods. Feature engineering-based QoS prediction methods are often developed based on time series prediction algorithms [24,25,26]. Yan et al. [24] considered the QoS prediction problem as a time series prediction problem and proposed an SVD-based ARIMA model for predicting multiple QoS values, which effectively improved the prediction accuracy of QoS. Hu et al. [25] proposed a personalized QoS prediction method, which combined a Kalman filter with an ARIMA model to provide the traditional ARIMA model with the ability to obtain feedback and correct the prediction. Keshavarzi et al. [26] proposed an online QoS time series prediction method combining clustering and minimum description length (MDL), which first clusters similar time series, and then the model generator uses MDL to obtain similar features from the time series.

Feature engineering-based methods often require targeted feature extraction methods designed for time series characteristics, which require skilled feature extraction theory and a high level of manual technical experience. In contrast, deep learning-based QoS prediction methods mainly use a deep neural network to obtain time series features, which has a low technical cost and significant effect improvement compared with traditional methods. Currently, dynamic QoS prediction algorithms based on deep learning frequently use previous QoS values as input, record their time series features, and then forecast QoS values at future points [27,28]. Jin et al. [29] divided the QoS prediction process into predictions based on historical time slices and predictions based on current time slices and proposed a two-stage method TWQP, which effectively solved the dynamic QoS prediction problem under different situations. Zhang et al. [30] proposed a multivariate time series QoS prediction approach that uses phase space reconstruction to translate multivariate historical data into a dynamic system and then uses a radial basis function (RBF) neural network modified by the Levenberg–Marquardt (LM) algorithm to execute a dynamic multi-step prediction. Zou et al. [31] provide a GRU-based deep neural network to mine the user and service temporal properties between users and services to predict unknown QoS. Additionally, they suggested an enhanced temporal characterization of users and services. However, there are two problems in the above research: firstly, the traditional deep learning-based QoS prediction algorithm fails to consider the real-time QoS values generated by the user when invoking the service as augmented information to be utilized in the prediction, making it impossible to further improve the model prediction accuracy. Secondly, the traditional deep learning models cannot fill in historical sparse QoS datasets during training, which affects the prediction accuracy of the models. A summary of the related work is shown in Table 1.

### 1.3. Main Contributions

To address the aforementioned issue, we propose MFDK, a three-part dynamic QoS prediction model: first, the user–service–time data is formed as a third-order tensor and decomposed into non-negative Tuckers to fill in the missing values in the tensor. Second, the tensor data is put into a CNN-BiLSTM deep learning model for training, and finally, the model predictions are adjusted by fusing QoS realistic observations through a Kalman filtering algorithm.

The main contributions of this paper are as follows.

- A new dynamic QoS prediction model called MFDK is built, which consists of three parts: missing data filling, deep learning model training, and Kalman filtering correction. The method can effectively solve the dynamic QoS prediction problem in the QoS sparse case.
- In comparison to typical deep learning models, a Kalman filter-based deep learning predictive value correction technique is developed, which has the benefit of more thoroughly merging the real-time QoS data induced by users and the model prediction data to increase the model prediction accuracy.
- Extensive experiments have been undertaken on the realistic dataset WS-DREAM. The experimental results indicate that our proposed framework is superior to the baseline model in terms of QoS forecast accuracy.

## 2. Preliminaries

### 2.1. Tucker Decomposition

**Figure 1:**
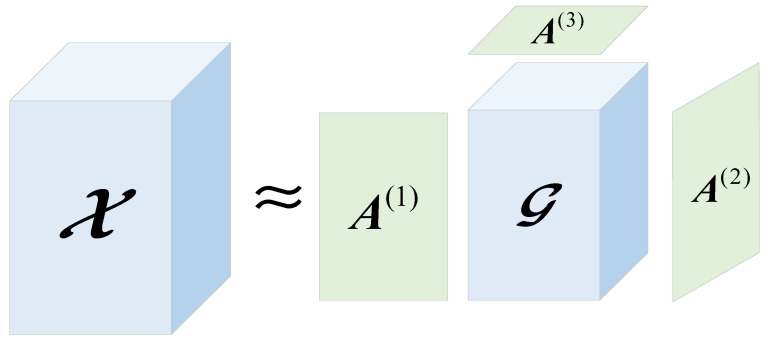
Schematic diagram of Tucker decomposition.

Tucker decomposition is one of the main tensor decomposition methods. The fundamental concept is to approximate the decomposition of the initial tensor as the product of the kernel tensor and the factor matrix. Using the third-order tensor, as an illustration, the decomposition has the form depicted in Figure 1.

Given a tensor $X\in R^{I_{1}\times I_{2}\times \cdots \times I_{N}}$, then the Tucker decomposition process of the tensor X can be expressed as:

(1) $$X\approx \hat{X}=G\times_{1}A^{\left(1\right)}\times_{2}A^{\left(2\right)}\times_{3}\cdots \times_{N}A^{\left(N\right)}$$

where $\hat{X}$ is the approximation tensor of X, $G\in R^{J_{1}\times J_{2}\times \cdots \times J_{N}}$ is the core tensor, and ${\left\{A^{\left(n\right)}\in R^{I_{n}\times J_{n}}\right\}}_{n=1}^{N}$ are the factor matrices. Solving the Tucker decomposition problem can be transformed into an optimization problem, as in Equation (2):

(2) $$\min{\left\|X-\hat{X}\right\|}_{F}^{2}$$

In this paper, the multiplicative updating algorithm is used to iteratively solve the problem. The objective function equation can be expressed as:

(3) $$\min{\left\|\left[X_{\left(n\right)}-A^{\left(n\right)}G_{\left(n\right)}(A_{⊗}^{\left(n\right)})\right]\right\|}_{F}^{2}$$

where $X_{\left(n\right)}$ is the mode-*n* matrix of tensor X and $G_{\left(n\right)}$ is the mode-*n* expansion matrix of the tensor G. We define $A_{⊗}^{\left(n\right)}=A^{\left(n+1\right)}⊗A^{\left(n+2\right)}\cdots ⊗A^{\left(N\right)}⊗A^{\left(1\right)}⊗A^{\left(2\right)}⊗\cdots A^{\left(n-1\right)}$, which collects Kronecker products of mode matrices except $A^{\left(n\right)}$, where ⊗ is the Kronecker product. Then the updated equations for the factor matrices and the core tensor are:

(4) $$A^{\left(n\right)}\leftarrow A^{\left(n\right)}*\frac{\left[X_{\left(n\right)}G_{A}^{\left(n\right)}{}^{T}\right]}{\left[A^{\left(n\right)}G_{A}^{\left(n\right)}G_{A}^{\left(n\right)}{}^{T}\right]}$$

(5) $$\begin{array}{c} G\leftarrow G*\frac{X\times_{1}A^{(1)T}\cdots \times_{n}A^{(N)T}}{G\times_{1}A^{(1)T}A^{\left(1\right)}\cdots \times_{n}A^{(N)T}A^{\left(N\right)}}\\ N=1,2,3. \end{array}$$

where $G_{A}^{\left(n\right)}=G_{\left(n\right)}(A_{⊗}^{\left(n\right)}{)}^{T}$, and ∗ is the Hadamard products. After performing the update, the model can be controlled to converge by setting the convergence threshold or by setting the number of iterations.

### 2.2. Kalman Filter

The Kalman filter algorithm mainly includes the time update equation and the state update equation, assuming the existence of a linear system with state and observation equations as follows:

(6) $$x_{k}=Ax_{k-1}+Bu_{k-1}+w_{k-1}$$

(7) $$z_{k}=Hx_{k}+v_{k}$$

where $x_{k}$ and $x_{k-1}$ are the states of the system at time *k* and *k* − 1, respectively, $u_{k-1}$ is the control variable at time *k* − 1, A and B are the state transfer matrix and the input state transfer matrix, respectively, $z_{k}$ is the observation at time *k*, H is the transformation matrix from state variable to observation variable, and $w_{k-1}$ and $v_{k}$ are Gaussian-distributed noise.

The time update equation for the Kalman filter is:

(8) $${\hat{x}}_{\bar{k}}=A{\hat{x}}_{k-1}+Bu_{k-1}$$

(9) $$P_{\bar{k}}=AP_{k-1}A^{T}+Q$$

where ${\hat{x}}_{\bar{k}}$ is the prior state estimate at time *k*, ${\hat{x}}_{k-1}$ is the posterior state estimate at time *k* − 1, $P_{\bar{k}}$ is the prior estimate covariance at time *k*, $P_{k-1}$ is the posterior estimate covariance at time *k* − 1, and Q is the state noise covariance. The state update equation is:

(10) $$K_{k}=\frac{P_{\bar{k}}H^{T}}{HP_{\bar{k}}H^{T}+R}$$

(11) $${\hat{x}}_{k}={\hat{x}}_{\bar{k}}+K_{k}(z_{k}-H{\hat{x}}_{\bar{k}})$$

(12) $$P_{k}=(I-K_{k}H)P_{\bar{k}}$$

where $K_{k}$ is the Kalman filter gain matrix, ${\hat{x}}_{k}$ is the a posteriori state estimate at time *k*, $P_{k}$ is the a posteriori estimated covariance at time *k* − 1, and $z_{k}$ is the observed value at time *k*. R is the observation noise covariance.

## 3. The Proposed Model

### 3.1. Description of the Problem

Before going into detail about the model proposed in this paper, a formal description of the problem solved in this paper is given.

**Figure 2:**
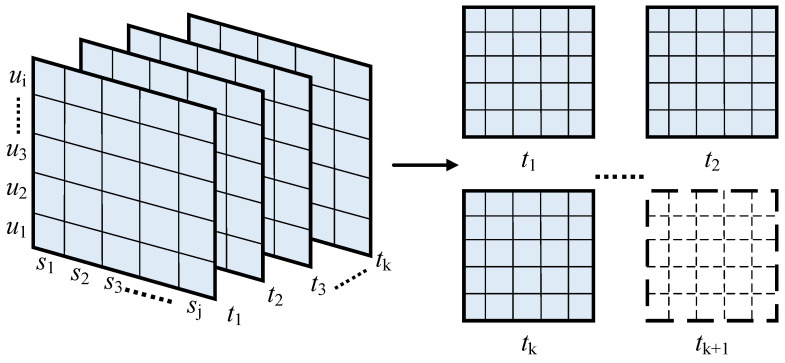
Users–service–time tensor.

Assuming that there exists a set of *I* users $U=\{u_{1},u_{2}\;\cdots \;u_{i}\cdots u_{I}\}$, a set of *J* services $S=\{s_{1},s_{2}\;\cdots \;s_{j}\cdots s_{J}\}$, and a set of *K* time slices $T=\{t_{1},t_{2}\;\cdots \;t_{k}\cdots t_{K}\}$, afterward, a third-order tensor $𝒚\in R^{I\times J\times K}$ can be constructed such that ${𝒚}_{ijk}$, the elements of 𝒚, represent the QoS values generated when the user *i* invokes the service *j* at time *k*. In real environments, the obtained QoS values are usually very sparse, which also makes the QoS tensor 𝒚 very sparse. As shown in Figure 2, the problem addressed in this paper is whether the QoS value at moment *k* + 1 can be predicted under the sparse case of 𝒚. To solve this problem, this paper proposes a dynamic QoS prediction method, MFDK, whose main process is described as follows.

**Figure 3:**
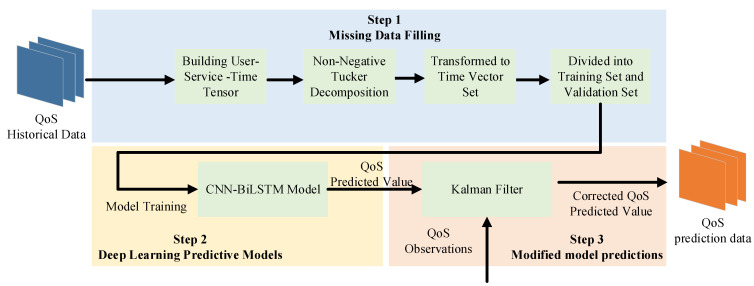
General structure of the MFDK model.

As seen in Figure 3, the historical QoS data is first converted into a user–service–time third-order tensor and then decomposed into a non-negative Tucker to fill in missing data. QoS predictions are obtained after dividing the full third-order tensor into a training and validation set and feeding it to a deep neural network for training. The final QoS predictions are obtained by combining the predictions produced by the deep neural network with the reality observations through the Kalman filter.

### 3.2. Missing Data Filling Based on Non-Negative Tucker Decomposition

The Tucker decomposition algorithm mentioned in Section 2.1 can serve to fill the missing values in the tensor to a certain extent; however, in practice, the missing values filled by Tucker decomposition can be negative, which has no practical significance in a QoS tensor with positive values. To improve the accuracy of the model prediction, this paper adds a non-negativity constraint to the Tucker decomposition, i.e., the missing data is filled by a non-negative Tucker decomposition. Thus, the objective function of the QoS tensor $𝒚\in R^{I\times J\times K}$ for non-negative Tucker decomposition can be expressed as:

(13) $$\begin{array}{c} \min_{n=1,2,3}{\left\|𝒚-\hat{𝒚}\right\|}_{F}^{2}\\ s.t.\;{\hat{𝒚}}_{ijk}\;>\;0,n=1,2,3 \end{array}$$

where ${\hat{𝒚}}_{ijk}$ is the element in the tensor to be predicted. Combined with the equations in Section 2.1, the non-negative Tucker decomposition Algorithm 1 can be summarized as follows:
**Algorithm 1:** Non-negative Tucker decomposition.Inputs: user-service-time tensor 𝒚, rank on each modal I, J, K Output: Tensor after filling sparse values 𝒚 1: **BEGIN** 2: Initialize the core tensor G and the factorization matrices $A^{\left(1\right)}$, $A^{\left(2\right)}$, and $A^{\left(3\right)}$ 3: **REPEAT** 4: **for** *n* **in** 3 **do**: 5: Iteratively update each factor matrix. $A^{\left(n\right)}\leftarrow A^{\left(n\right)}*\frac{\left[X_{\left(n\right)}G_{A}^{\left(n\right)}{}^{T}\right]}{\left[A^{\left(n\right)}G_{A}^{\left(n\right)}G_{A}^{\left(n\right)}{}^{T}\right]}$ 6: end for 7: Updating the core tensor. $G\leftarrow G*\frac{𝒚\times_{1}A^{(1)T}\times_{2}A^{(2)T}\times_{3}A^{(3)T}}{G\times_{1}A^{(1)T}A^{\left(1\right)}\times_{2}A^{(2)T}A^{\left(2\right)}\times_{3}A^{(3)T}A^{\left(3\right)}}$ 8: Calculate the updated tensor.
$\hat{𝒚}=G\times_{1}A^{\left(1\right)}\times_{2}A^{\left(2\right)}\times_{3}A^{\left(3\right)}$
9: Tensor iterative update.
$𝒚\leftarrow \hat{𝒚}$
10: until the error converges or the maximum number of iterations is reached 11: **END**


### 3.3. CNN-BiLSTM Based Time Series Prediction Model

#### 3.3.1. Training Dataset Construction

**Figure 4:**
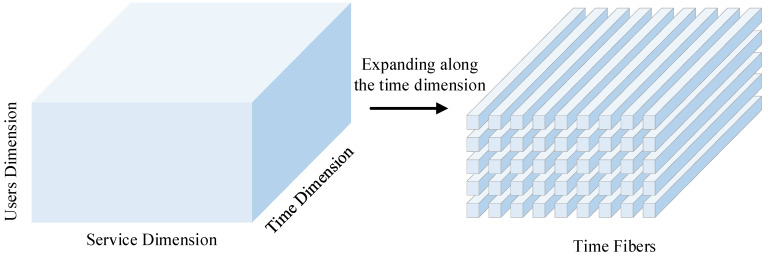
Schematic representation of tensor expansion into fiber patterns.

After filling in the historical data, a CNN-BiLSTM neural network model is constructed in this paper to train on the historical data. Prior to training, the user–service–time tensor needs to be constructed as training data that can be fed into the neural network. To make predictions of QoS values for future time slices, the tensor is expanded into fibers forming along the time dimension, as in Figure 4.

In this paper, the first K−1 time slices of each vector after unfolding are used as model training data and the Kth step is predicted to achieve QoS prediction for future time slices.

#### 3.3.2. Convolutional Neural Network

In this paper, the local features of QoS time series are extracted by convolution neural network. The advantage of CNN lies in its ability of local feature extraction and parameter sharing. Convolutional kernels are used by the convolutional layer to convolve local regions of the QoS time series in order to create corresponding features and reduce the risk of over-fitting through the parameter sharing of the convolution kernel. The process of convolution kernel convolution operation is as follows:

(14) $$F_{t}=\varphi (W_{t}x_{t-1}+b_{t})$$

where $F_{t}$ represents the result of the convolution operation of the *t*-th convolution kernel; φ represents the nonlinear activation function; and $W_{t}$, $x_{t-1}$, and $b_{t}$ represent the filter kernel, the input of the CNN layer, and bias term of the *t*-th convolution kernel.

The output of convolution layer is shown as the connection of all convolution kernel calculation results, which is shown as:

(15) $$output=[F_{1},F_{2},\cdots ,F_{n}]$$

where n represents the maximum number of convolution kernels in the convolution layer; output represents the final output of the convolution layer.

#### 3.3.3. Bidirectional Long Short-Term Memory Neural Network

**Figure 5:**
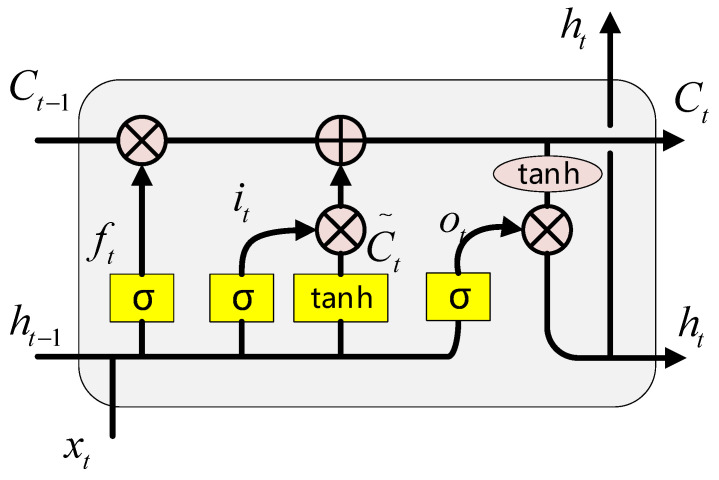
An LSTM unit structure.

In this paper, a BiLSTM layer is added after the CNN layer to obtain the global characteristics of time series. BiLSTM is a unique RNN structure. It is composed of a two-layer LSTM network, which can obtain sequence features from two directions. The core structure of the LSTM consists of an input gate, a forgetting gate, a memory unit, and an output gate. Input information enters the LSTM unit through the input gate, determines the information to be retained and forgotten through the forgetting gate, and finally outputs the information through the output gate. Through such a unique gate structure, LSTM alleviates the problems of gradient disappearance and gradient explosion in traditional RNN networks. The structure of the LSTM unit is shown in Figure 5.

The calculation process of forgetting gate is as follows:

(16) $$f_{t}=\sigma (W_{f}\cdot [h_{t-1},x_{t}]+b_{f})$$

where $f_{t}$ is the output of the forgetting gate; $x_{t}$ represents the input feature sequence; and $h_{t-1}$ represents the output sequence of the previous time. $W_{f}$ is the weight matrix of the forgetting gate; $b_{f}$ represents the offset matrix; and σ is the sigmoid activation function, whose expression is:

(17) $$\sigma (x)=\frac{1}{1+e^{-x}}$$

The calculation process of input gate and memory unit is as follows:

(18) $$i_{t}=\sigma (W_{i}\cdot [h_{t-1},x_{t}]+b_{i})$$

(19) $$\tilde{C_{t}}=\tanh(W_{c}\cdot [h_{t-1},x_{t}]+b_{c})$$

(20) $$C_{t}=f_{t}*C_{t-1}+i_{t}*\tilde{C_{t}}$$

where $h_{t-1}$ and $x_{t}$ generate the intermediate variable $\tilde{C_{t}}$ through the activation function tanh, and at the same time, $i_{t}$, as the output of the input gate, calculates the value of the memory state $C_{t}$ together with $f_{t}$; $W_{i}$ and $W_{c}$ are weight matrices; $b_{i}$ and $b_{c}$ are offset matrices.

**Figure 6:**
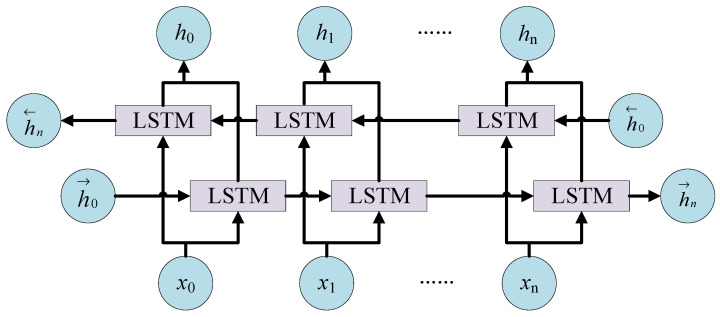
Bidirectional long short-term memory (BiLSTM) neural network structure.

Finally, the output information of the LSTM unit is determined through the output gate, whose expression is:

(21) $$o_{t}=\sigma (W_{o}\cdot [h_{t-1},x_{t}]+b_{o})$$

(22) $$h_{t}=o_{t}*\tanh(C_{t})$$

where $o_{t}$ represents the output of the output gate, which together with $C_{t}$ determines the short-term memory $h_{t}$ of the LSTM unit at time *t*. The BiLSTM network used in this paper consists of the above LSTM units. Its composition is shown in Figure 6.

The BiLSTM network is composed of forward LSTM layer and reverse LSTM layer, whose expression is:

(23) $$h_{t}=[{\vec{h}}_{n},{\overset{\leftarrow }{h}}_{n}]$$

where ${\vec{h}}_{n}$ is the calculation result of the forward LSTM network, ${\overset{\leftarrow }{h}}_{n}$ is the calculation result of the reverse LSTM network, and $h_{t}$ is the final output of the BiLSTM network. In this way, BiLSTM can well obtain the global feature information of time series.

#### 3.3.4. The Overall Structure of The Model

In order to fully obtain the temporal characteristics of historical QoS data, a neural network model based on CNN and BiLSTM is adopted in this paper. Due to its circular structure, a recurrent neural network (RNN) has good advantages in capturing the characteristics of time series. However, the traditional RNN neural network faces the challenge of gradient disappearance and gradient explosion. In order to fully obtain the characteristics of historical QoS data and improve the universality of the network, this paper uses a BiLSTM neural network to obtain the time series characteristics of historical QoS data. The structure of a BiLSTM neural network can obtain features from the forward and reverse of time series, which greatly improves the feature capture ability of the model while alleviating the problems of gradient disappearance and gradient explosion. On this basis, the CNN layer is added to the BiLSTM model to enhance the model’s ability to obtain local features of time series.

**Figure 7:**
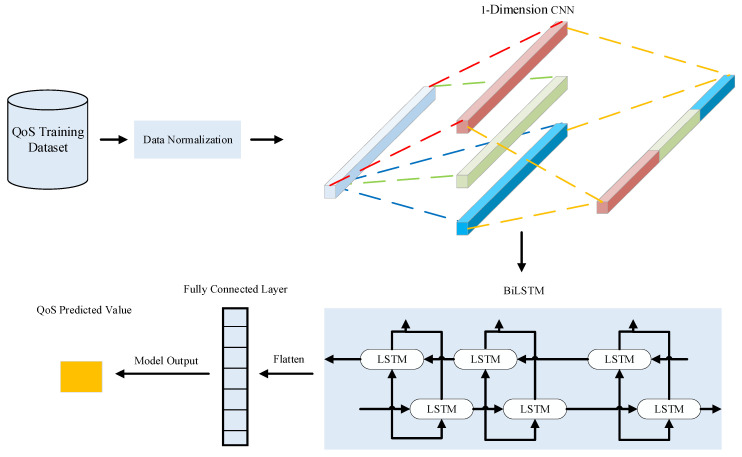
CNN-BiLSTM Neural Network Structure.

As shown in Figure 7, since the normalized data will make the model complete convergence faster, the QoS data will be normalized first. In this paper, we use the maximum-minimum normalization to operate on the data, and the calculation formula is:

(24) $$X_{norm}=\frac{X-X_{\min}}{X_{\max}-X_{\min}}$$

where X is the original data in the time series, $X_{\min}$ is the minimum value in the time series, $X_{\max}$ is the maximum value in the time series, and $X_{norm}$ is the normalized data.

In this paper, the local features in the time series are first extracted using a one-dimensional CNN layer. For the features of the QoS time series, this model uses a convolutional layer with a window length of 5 and a total of 64 one-dimensional convolutional kernels to extract features from the time series.

The main purpose of the BiLSTM neural network is to further learn the overall temporal characteristics of the time series context based on the local features acquired by the CNN network. The model uses BiLSTM layers containing 64 LSTM units per layer.

After the output of the BiLSTM layer, the model is expanded into a one-dimensional sequence through the flatten layer and a single element is output as the next moment QoS prediction through the fully connected layer. The Adams optimizer algorithm was used to continuously update the model parameters by monitoring the training error “MAE”. The batch size was 32, and the maximum number of training epochs was 200.

### 3.4. Kalman Filter-Based Deep Learning Predictive Value Correction Algorithm

#### 3.4.1. Derivation of the Algorithm Formula

The application of deep learning in the time series prediction process is often based on the prediction results of the model itself as the final prediction value, while in a realistic QoS prediction application environment, real-time QoS observations will also be constantly available during the calculation of deep learning prediction values. If the real-time QoS observations can be combined with the prediction values of deep neural networks, it will be possible to improve the prediction accuracy based on the deep neural network prediction. Based on the above ideas, this paper proposes a Kalman filter-based correction algorithm for deep learning prediction values. Based on the Kalman filter formula in Section 1.2, this algorithm treats the predicted values of the deep neural network as the next predicted values of the Kalman filter, and the predicted QoS values are all in elemental form. Then the state equation will be transformable into:

(25) $${\hat{x}}_{\bar{k}}=net_{\bar{k}}$$

(26) $$P_{\bar{k}}=loss_{k-1}+Q$$

where $net_{\bar{k}}$ is the prediction value of the neural network at moment *k* − 1 and $loss_{k-1}$ is the prediction error of the neural network at moment *k* − 1. To calculate the error of the prediction process and quantify the value of $loss_{k-1}$, assume that there exists a time-variable state transfer variable $A_{k-1}$ such that:

(27) $${\hat{x}}_{\bar{k}}=net_{\bar{k}}=A_{k-1}net_{\bar{k}-1}$$

Then Equation (9) can be transformed into:

(28) $$P_{\bar{k}}=P_{k-1}A_{k-1}{}^{2}+Q$$

where $A_{k-1}=\frac{net_{\bar{k}}}{net_{\bar{k}-1}}$; then, substituting Equations (21) and (22) into the state update equation gives:

(29) $$K_{k}=\frac{P_{\bar{k}}}{P_{\bar{k}}+R}$$

(30) $$Knet_{k}=net_{\bar{k}}+K_{k}(QoS_{k}-net_{\bar{k}})$$

where $QoS_{k}$ is the observed value at moment *k* and $Knet_{k}$ is the predicted value at moment *k* corrected by the Kalman filter.

In summary, the Algorithm 2 is summarized as follows.
**Algorithm 2:** Kalman filter-based deep learning predictive value correction algorithm.Inputs: $net_{\bar{k}}$:predicted value of the model at moment *k*,               $QoS_{k}$:real-time QoS observations at moment *k* Output: $Knet_{k}$:Kalman filtered predictions at moment *k* 1: **BEGIN** 2: Kalman filter initialization: Q, R, $P_{0}$, $net_{1}$, k=2 3: **REPEAT** 4: Set the model predictions to the Kalman filter one-step predictions.
${\hat{x}}_{\bar{k}}=net_{\bar{k}}$ 5: Calculate the state transfer variables $A_{k-1}$
:
$A_{k-1}=\frac{net_{\bar{k}}}{net_{\bar{k}-1}}$ 6: Calculate the covariance from $A_{k-1}$:
$P_{\bar{k}}=P_{k-1}A_{k-1}{}^{2}+Q$ 7: Calculate the Kalman filter gain $K_{k}$:
$K_{k}=\frac{P_{\bar{k}}}{P_{\bar{k}}+R}$ 8: Calculation of Kalman filter corrected model predictions:
$Knet_{k}=net_{\bar{k}}+K_{k}(QoS_{k}-net_{\bar{k}})$ 9: Calculation of post-prediction covariance:
$P_{k}=(1-K_{k})P_{\bar{k}}$ 10: k=k+1 11: until end of algorithm 12: **END**


#### 3.4.2. Optimization Strategies in the Face of Sparse Observations

Algorithm 2 achieves an effective combination of model predictions and real-time QoS observations. However, in practice, real-time QoS observations are also sparse, and missing observations added to the Kalman filtering process may lead to an increase in algorithm error. In this paper, an algorithm optimization strategy is proposed for the case of sparse observations. Algorithm 3 is the optimized algorithm, and the specific contents are as follows
**Algorithm 3:** Kalman filter-based deep learning prediction correction algorithm under sparse observations.Inputs: $net_{\bar{k}}$:predicted value of the model at moment *k*,                $QoS_{k}$:real-time QoS observations at moment *k* Output: $Knet_{k}$:Kalman filtered predictions at moment *k* 1: **BEGIN** 2: Kalman filter initialization:Q, R, $P_{0}$, $net_{1}$, k=2 3: **REPEAT** 4: Set the model predictions to the Kalman filter one-step predictions: ${\hat{x}}_{\bar{k}}=net_{\bar{k}}$ 5: Calculate the state transfer variables $A_{k-1}$
:
$A_{k-1}=\frac{net_{\bar{k}}}{net_{\bar{k}-1}}$ 6: Calculate the covariance from $A_{k-1}$:
$P_{\bar{k}}=P_{k-1}A_{k-1}{}^{2}+Q$ 7: Calculate the Kalman filter gain $K_{k}$:
$K_{k}=\frac{P_{\bar{k}}}{P_{\bar{k}}+R}$ 8: Determine if a QoS observation is missing. **if**
$QoS_{k}\neq 0$ 9: Calculation of Kalman filter corrected model predictions. $Knet_{k}=net_{\bar{k}}+K_{k}(QoS_{k}-net_{\bar{k}})$ 10: **else** 11: Use the model predictions as Kalman filter corrections. $Knet_{k}=net_{\bar{k}}$ 12: **end if** 13: Calculation of post-prediction covariance. $P_{k}=(1-K_{k})P_{\bar{k}}$ 14: k=k+1 15: until the end of the algorithm 16: **END**


## 4. Experimental Results and Analysis

### 4.1. Preparation

#### 4.1.1. Data Set and Experimental Environment

This paper conducts experiments on a response time dataset from the WS-DREAM-dataset 2 dataset, which is widely used in the field of QoS prediction. It contains response time data generated by 142 users interacting with 4500 web services over 64 time slices. The experiments in this paper construct the data in the dataset as a tensor and then perform missing value filling, expand the tensor fibrillation into a time series vector according to the method in Section 3.3.1, and keep the first 63 time slices in the vector as historical data for training and predicting the outcome of the next time slice. The value of the 64th time slice is taken as the true value. When dividing the dataset, 85% of the vectors were used as the deep learning model training set and the remaining 15% were used as model validation for training.

This experiment is based on the Intel (R) core (TM) i7-10875h CPU at 2.30 GHz. On the Windows platform of the processor, the NVIDIA Geforce GTX 2060 graphics processor and CUDA version 11.1.114 are used for the training process of deep learning. All codes are programmed through the PyCharm platform.

#### 4.1.2. Evaluation Indicators

The mean absolute error (MAE) and root mean square error (RMSE) are used in the experiments in this paper as evaluation metrics in the dynamic QoS prediction process. The MAE and RMSE are defined as:

(31) $$\mathrm{MAE}=\frac{\sum_{i=1}^{n}\left|real_{i}-predicted_{i}\right|}{n}$$

(32) $$\mathrm{RMSE}=\sqrt{\frac{\sum_{i=1}^{n}{\left(real_{i}-predicted_{i}\right)}^{2}}{n}}$$

where n is the number of predicted QoS values, and $real_{i}$ and $predicted_{i}$ are the true and predicted values, respectively. The smaller the MAE and RMSE values, the greater the prediction effect of the relevant model.

### 4.2. Model Comparison Experiments

This section compares the MFDK algorithm proposed in this paper with existing time series prediction algorithms and time-aware QoS prediction-based algorithms.

One of them is the ARIMA [24] algorithm (Autoregressive Integrated Moving Average model, ARIMA), which is a classical time series prediction algorithm that performs model prediction by smoothed time series parameters obtained after differencing.

TCN [32] (Temporal Convolutional Network, TCN) is a type of convolutional neural network that uses causal convolution and dilated convolution to enable the convolutional neural network to handle time series prediction problems. It is a new model that outperforms traditional neural networks such as LSTM and RNN in prediction performance.

WSPred [33] is a time series aware QoS prediction algorithm based on tensor decomposition, which predicts missing values by performing a third-order tensor decomposition on the user–service–time tensor.

CLUS [34] clusters the user service time tensor into different clusters through the K-means algorithm and uses the similarity relationship within the cluster to predict QoS.

The PMF [35] (Probabilistic Matrix Factorization) method performs QoS prediction by means of probabilistic matrix factorization.

To test the adaptability of the different methods in a realistic QoS sparse environment, we randomly removed data from the tensor to control the tensor density; e.g., a tensor with a tensor density of 0.1 represents a tensor where we randomly removed 90% of the elements of the original tensor. We finally compared the RMSE and MAE metrics of the different prediction methods for tensor densities equal to 0.1, 0.15, 0.2, 0.25, and 0.3. The final experimental results are shown in Table 2.

The experimental results demonstrate that the prediction accuracy of each model generally improves as the tensor density increases. The RMSE and MAE indices of the model proposed in this paper are lower than those of the comparison models under various tensor densities, indicating that the MFDK model has superior QoS prediction capability. Specifically, the ARIMA model has limited capacity to capture sparse time series characteristics and cannot fill in missing data, making it hard to construct an appropriate prediction model for sparse data. The TCN algorithm is a deep learning method that captures the features of time series data excellently. However, the capacity for generalization and prediction of a TCN network trained on sparse data would be considerably diminished. This further demonstrates the significance of data filling for historical data. The PMF, CLUS, and WSPred algorithm models have better prediction capabilities compared to the ARIMA and TCN models, with their RMSE metrics decreasing by averages of 53.2%, 23.5%, and 34.6%, respectively, and their MAE metrics decreasing by averages of 28.6%, 17.3%, and 12.1%, respectively. This is due to the capacity of time-aware QoS prediction algorithms to fill in sparse data. They are better at adapting to sparse data than ARIMA and TCN algorithms. Among these, the WSpred algorithm outperforms PMF and CLUS in terms of prediction accuracy. This is because the WSpred algorithm iteratively predicts missing values in a gradient descent manner using a third-order tensor containing time series relationships, and the prediction accuracy is greater. However, the aforementioned model lacks the combination of real-time QoS observations, and its prediction impact is still insufficient when compared to the MFDK model. To summarize, the MFDK model with sparse data filling and real-time QoS observations has greatly increased QoS prediction accuracy when compared to baseline QoS prediction approaches.

### 4.3. Model Ablation Experiments

**Figure 8:**
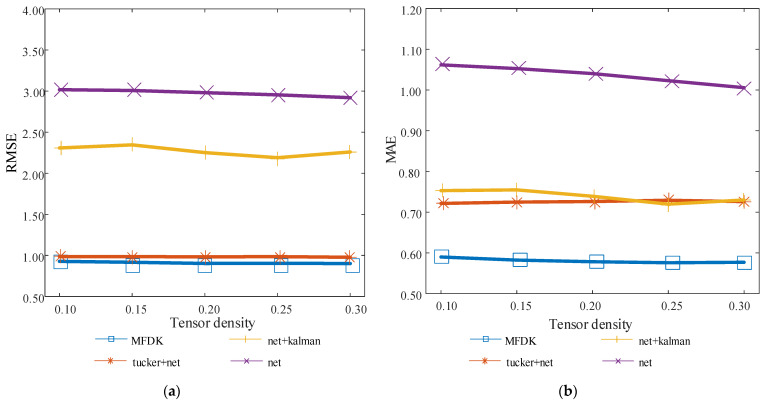
Results of model ablation experiments. (**a**) Variation of RMSE at different tensor densities in the ablation experiment; (**b**) variation of MAE at different tensor densities in the ablation experiment.

The experiments in this part are designed to check the functions of the various components of the model provided in this study to further evaluate the model’s rationality and predictive capacity. The Tucker + net and net + Kalman models are created by deleting the Kalman filter correction and non-negative matrix decomposition parts of the MFDK model, respectively, while the net model merely keeps the neural network prediction element of the MFDK model. The outcomes of their experiments are displayed in Figure 8.

Based on the experimental findings, it is evident that as tensor density increases, the overall prediction accuracy of each model improves. Among these, the MFDK model achieves better experimental results, with average reductions of 69.4% and 44.0% in RMSE and Mae indicators, respectively, compared with net. From the experimental results of the Tucker + net model and net + Kalman model, it can be seen that, relative to the net model, the RMSE and MAE of the former decreased by averages of 66.9% and 30.0%, while the RMSE and MAE of the latter decreased by averages of 23.7% and 28.7%, which were less than the former. This is because the non-negative matrix decomposition predicts the missing QoS data, fills the sparsity of the training data, considerably enhances the capacity of the neural network to forecast, and greatly increases the overall prediction accuracy of the model. The experimental findings in this part demonstrate that both non-negative matrix factorization and the Kalman filter have significantly enhanced the neural network’s prediction outcomes, proving that the MFDK model has good performance.

### 4.4. Effect of Observation Sparsity on Model Performance

**Figure 9:**
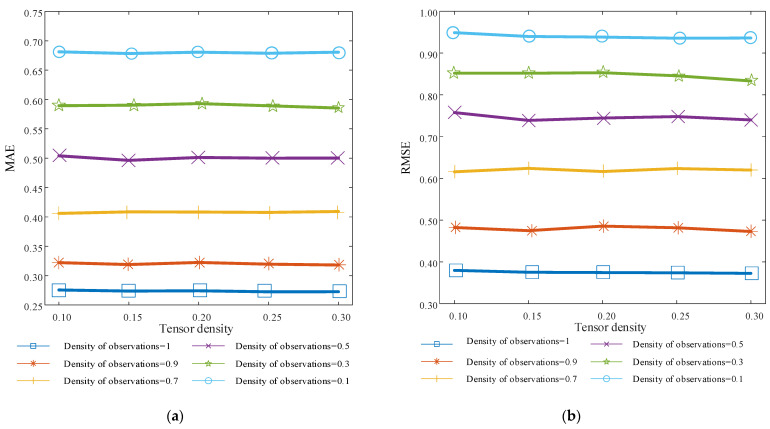
Experimental results on the variation of the sparsity of the observations. (**a**) MAE variation for different tensor densities in the observation variation; (**b**) RMSE variation at different tensor densities in the observation variation.

In the real environment, the real-time observation value of the QoS value is also sparse. This section mainly tests the adaptability of MFDK model to the sparsity of different observations and its applicability in the real environment. This experiment verifies the prediction performance of the model under different tensor densities when the observation value densities are 1, 0.9, 0.7, 0.5, 0.3, and 0.1. The experimental results are shown in Figure 9 below.

It can be seen from the experimental results that the overall prediction accuracy of the model shows an upward trend with the increase of tensor density, and the prediction accuracy of the model is also rising with the increase of observation density. Specifically, starting from the observation density of 0.1, the RMSE and MAE of the model will decrease by 16.6% on average every time the observation density increases by 0.2. When the observation density is one, that is, the observation value has no sparsity, the prediction effect of the model reaches the best, and the RMSE and MAE are 0.3752 and 0.2736 on average. It can be further concluded from the experiment that the MFDK model can well adapt to the observed values under different sparsities. At the same time, the predicted values of the model can be modified by integrating the observed values with the Kalman filter algorithm, which can effectively improve the prediction accuracy of the model. The lower the sparsity of the observed values, the better the prediction effect of the model.

### 4.5. Effect of Kalman Filter Parameters on Model Performance

The experiment in this section mainly discusses the influence of changes in state noise covariance *Q* and observation noise covariance *R* in Kalman filter parameters on the prediction performance of the model. *Q* and *R* are the initial parameters in the Kalman filter algorithm, and their values represent the confidence of the Kalman filter to the predicted value of the model and the actual observed value, respectively, thus affecting the correction ability of the predicted value of the model. It should be noted that in the actual calculation process, *Q* and *R* do not affect the Kalman filter process alone. According to Formulas (22) and (23), *Q* and *R* jointly determine the calculation process of Kalman filter gain. Therefore, this experiment will discuss the change of the prediction ability of the model under the conditions of Q∈[0,20] and R∈[0,20], when the observed value density is 0.5 and the tensor densities are 0.1, 0.3, and 0.5, respectively. The experimental results are as follows.

**Figure 10:**
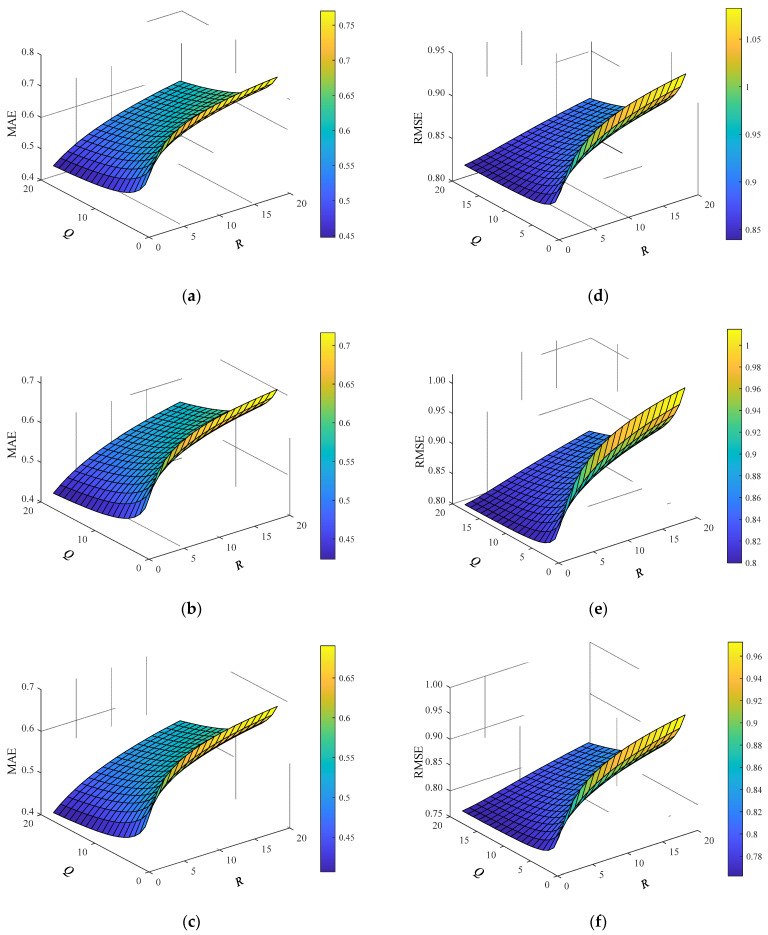
Experimental results of Kalman filter parameter variations. (**a**) Variation of MAE at tensor density of 0.1; (**b**) variation of MAE at tensor density of 0.3; (**c**) variation of MAE at tensor density of 0.5; (**d**) variation of PMSE at tensor density of 0.1; (**e**) variation of PMSE at tensor density of 0.3; (**f**) variation of PMSE at tensor density of 0.5.

As shown in Figure 10, the three-dimensional chart is composed of the *x*-axis as the state noise covariance *Q*, the *y*-axis as the observation noise covariance *R,* and the *z*-axis as the model evaluation indicators RMSE and MAE, respectively. From the chart, it can be concluded that the changes of *R* and *Q* values have an important impact on the prediction accuracy of the model. Specifically, with the continuous improvement of tensor density, the overall prediction accuracy of the model is also rising. Further, in each group of experiments, when the *R* value decreases and *Q* value increases, RMSE and MAE decline as a whole, and the prediction ability of the model increases; This is because with the decrease of *R* value and the increase of *Q* value, the gain value of Kalman filter will increase, the ability of Kalman filter to correct the predicted value of the model will be enhanced, and the prediction accuracy will also be improved. From this analysis, we can draw a conclusion: the changes of Kalman filter parameters *R* and *Q* have a significant impact on the prediction accuracy of the model. When initializing Kalman filter parameters, increasing *Q* and decreasing *R* will further improve the prediction accuracy of the model.

## 5. Conclusions

This study introduces MFDK, a novel dynamic QoS prediction approach. The approach is divided into three sections. To begin, non-negative Tucker decomposition is used to fill in the sparse values in historical data; after that, the historical QoS data is transformed into training data, which is then transferred to the CNN-BiLSTM deep learning model we built for training, and the QoS value of the future time slice is predicted. Finally, the prediction values are adjusted using the real-time QoS method introduced in this research, which combines the real observation values with the Kalman filter to obtain more accurate QoS prediction data. The MFDK model successfully handles the challenges of historical QoS data filling and real-time QoS data fusion via trials. Experiments on the WS-dream dataset revealed that the MFDK model outperforms the classic dynamic QoS prediction approach.

In the future work, the development direction of the MFDK model will be investigated in two aspects: on the one hand, it will continue to improve the filling ability of sparse QoS data. Information such as user geographic location and user–service–similarity relationships can be combined to make the historical data filling results of the MFDK model more accurate; on the other hand, the performance of the deep learning network will be improved so that the ability of the new neural network to capture time series features can be enhanced.

## Figures and Tables

**Table 1:** Comparison of methods and results of research in related work.

Algorithm Category		Approach	Prediction Accuracy of QoS	References
Static QoS prediction algorithm	Neighborhood-based CF algorithm	A CF-based approach for mining the similarity of users.	Outperforms common collaborative filtering algorithms and average prediction algorithms in terms of response time, availability, and latency	Shao et al. [19]
		WSRec: a improved CF-based approach for combining the traditional user-based and item-based CF methods.	Better than UMAEN, IMEAN, UPCC, IPCC algorithms in terms of response time and failure rate	Zheng et al. [20]
	Model-based CF algorithm	JDNMFL: A method based on a combination of matrix decomposition and neural networks, including multi-source feature extraction and feature interaction learning.	Better than UPCC, IPCC, UIPCC, PMF, FM algorithms in terms of response time and throughput	Xia et al. [21]
		NDMF: A method integrates user neighborhood selected by a collaborative way into an enhanced matrix factorization model via deep neural network.	Outperforms the 12 baseline models in the article in terms of response time and throughput	Zou et al. [22]
		AMF: A method combines probabilistic matrix decomposition and neural attention networks for QoS prediction.	Outperforms the 8 baseline models in the article in terms of Normalized Discounted Cumulative Gain (NDCG) and Mean average precision (MAP)	Nguyen et al. [23]
Dynamic QoS prediction algorithm	Feature engineering-based algorithm	A method combines a truncated singular value decomposition (SVD) and a classical ARIMA model.	Better than UPCC, IPCC, SerRec algorithms in terms of response time and throughput	Yan et al. [24]
		A method combines Kalman filtering and classical ARIMA model. Afterwards, personalized QoS prediction is achieved by an modified neighborhood-based CF algorithm.	Better than ARIMA, WSRec algorithms in terms of response time and throughput	Hu et al. [25]
		A method combines time series clustering, minimum description length and dynamic time warping similarity. Afterwards, the most appropriate service quality prediction scheme is provided to the user via multi-cloud.	Better than UPCC, IPCC, combined UPCC and IPCC, LASSO algorithms in terms of response time and throughput	Keshavarzi et al. [26]
	Deep learning-based QoS prediction algorithm	TWQP: A two-stage QoS prediction method that performs predictions in the historical time slice and the current time slice, respectively.	Outperforms the 6 baseline models in the article in terms of response time and throughput	Jin et al. [29]
		MulA-LMRBF: A method to input historical QoS data using phase-space reconstruction method, afterwards implementing dynamic multi-step prediction by RBF neural network improved by Levenberg–Marquardt algorithm.	Outperforms the 5 baseline models in the article in terms of response time and throughput	Zhang et al. [30]
		DeepTSQP: A method propose a deep neural network with gated recurrent units (GRU), learning, and mining temporal features among users and services.	Outperforms the 9 baseline models in the article in terms of response time and throughput	Zou et al. [31]

**Table 2:** Results of model comparison experiments.

Forecasting Methodology	Tensor Density									
	0.1		0.15		0.2		0.25		0.3	
	RMSE	MAE	RMSE	MAE	RMSE	MAE	RMSE	MAE	RMSE	MAE
ARIMA [24]	2.9209	1.0471	2.8388	1.0225	2.7578	0.9866	2.6186	0.9376	2.5119	0.9008
TCN [32]	3.0182	1.1188	2.9754	1.0966	2.9146	1.0773	2.9422	1.0682	2.8556	1.0502
WSPred [33]	1.7878	0.7684	1.7737	0.7563	1.7864	0.7653	1.7708	0.7512	1.7921	0.7638
CLUS [34]	2.2625	0.8858	2.2494	0.8557	2.2168	0.8296	2.1782	0.8082	2.1434	0.7926
PMF [35]	2.2441	0.9336	2.0951	0.8951	1.9961	0.8667	1.9271	0.8448	1.8773	0.8271
**MFDK**	**0.9282**	**0.5901**	**0.9169**	**0.5825**	**0.9019**	**0.5784**	**0.9042**	**0.5759**	**0.9006**	**0.5770**

## Data Availability

The dataset used in this study is derived from the open dataset of Web services research, wsdream-dataset2, publicly available at the Chinese University of Hong Kong. https://github.com/wsdream/wsdream-dataset (accessed on 18 June 2022). The experimental results of CLUS, WSPred, and PMF are cited in Jieming Zhu, Pinjia He, Zibin Zheng, and Michael R. Lyu, “Benchmarking and Improving QoS Prediction Approaches for Web Service Recommendation”, available online: http://wsdream.github.io/WSRec (accessed on 18 June 2022).
